# Pathophysiology and Treatment of Pruritus in Elderly

**DOI:** 10.3390/ijms22010174

**Published:** 2020-12-26

**Authors:** Bo Young Chung, Ji Young Um, Jin Cheol Kim, Seok Young Kang, Chun Wook Park, Hye One Kim

**Affiliations:** Department of Dermatology, Kangnam Sacred Heart Hospital, Hallym University, Seoul KS013, Korea; victoryby@naver.com (B.Y.C.); ujy0402@hanmail.net (J.Y.U.); aiekfne@naver.com (J.C.K.); tjdjrdud@naver.com (S.Y.K.); dermap@hanmail.net (C.W.P.)

**Keywords:** elderly, ion channel, itch, neurotransmission pathophysiology of itch, pruritogen, senile pruritus, treatment of itch

## Abstract

Pruritus is a relatively common symptom that anyone can experience at any point in their life and is more common in the elderly. Pruritus in elderly can be defined as chronic pruritus in a person over 65 years old. The pathophysiology of pruritus in elderly is still unclear, and the quality of life is reduced. Generally, itch can be clinically classified into six types: Itch caused by systemic diseases, itch caused by skin diseases, neuropathic pruritus, psychogenic pruritus, pruritus with multiple factors, and from unknown causes. Senile pruritus can be defined as a chronic pruritus of unknown origin in elderly people. Various neuronal mediators, signaling mechanisms at neuronal terminals, central and peripheral neurotransmission pathways, and neuronal sensitizations are included in the processes causing itch. A variety of therapies are used and several novel drugs are being developed to relieve itch, including systemic and topical treatments.

## 1. Introduction

Pruritus is a relatively common symptom that anyone can experience at any point in their life especially in elderly population. Pruritus in elderly can be defined as idiopathic chronic pruritus in a person over 65 years old. Pruritus may present with or without skin lesions. In previous reports, the prevalence of pruritus in elderly patients was 11–78% [1,2,3]. The etiology of pruritus in elderly is unknown; however, many elderly people also complain pruritus caused by various specific causes not only from xerosis and dermatologic diseases but also from several systemic disorders [4]. Chronic itch markedly worsens the quality of life of elderly patients. Chronic pruritus can have a significant effect on quality of life. In most elderly people, pruritus is not just an occasional problem; it can induce debilitating effects, such as irritation and sleep impairment, which can result in clinical depression. In fact, most patients with chronic pruritus can become so depressed that they would rather live a shorter life free of symptoms than a longer life with pruritus that the detrimental effect of chronic pruritus on quality of life is comparable with that of chronic pain [5,6].

## 2. Classification and Causes of Itch in Elderly

Itch may be caused secondarily by skin diseases and systemic diseases in the elderly. Classification of diseases that provoke itch exhibits the characteristic clinical features [7] (Table 1).

Clinically, itch can be divided into six types: itch caused by systemic diseases, itch caused by skin diseases, neuropathic pruritus, psychogenic pruritus, pruritus with multiple factors, and from unknown causes [8]. Senile pruritus is an idiopathic itching without a primary rash in elderly [9]. Identifying the underlying causes of chronic pruritus tends to be more difficult in older patients. In many cases, it is possible to identify some possible dermatological and non-dermatological diseases that cause itch regardless of the apparent time between the onset of symptoms and the onset of possible causes. In addition, there are often multiple drugs that ultimately contribute to pruritus by inducing side effects or drug associated eczematous skin lesions. Therefore, diagnostic work-up including patient history and laboratory tests is required for accurate diagnosis. For chronic pruritus of inflamed or excoriated skin lesions, a skin biopsy for histological or immunofluorescence testing may be required.

Skin diseases that induce itch mainly include eczematous dermatitis, hives, food allergies, insect bites, and scabies. In addition to xerosis, multiple dermatological conditions are related to chronic itch in the elderly with higher intensity [10]. Seborrhoeic dermatitis (SD) is a chronic and particularly common skin manifestation characterized by overlying adherent, greasy scales. SD predominantly affects oily areas of the body, such as the scalp, periauricular area, nasolabial folds, cheeks, sternal area and interscapular areas and may also affect other body folds [11]. Within the elderly population, one-third of the geriatric population suffers from SD, which is associated with localized itch [11,12].

Nummular eczema (NE) is an extremely pruritic, inflammatory skin disease found in elderly patients and can be considered a late-onset form of atopic dermatitis [13,14]. The degree of itching varies depending on the affected area and how sensitive the patient is. Severe scratching or rubbing to eliminate the itch without proper treatment of causal diseases may result in skin damage, such as scratches, abrasions, erythema, lichenification, ulcers, and pigmentation of the skin.

If an itching sensation occurs on the skin due to systemic diseases, then kidney disease, liver disease, gastrointestinal disease, or cancer could be the problem. Additional comprehensive laboratory tests are needed to rule out associated systemic disorders such as diabetes, renal dysfunction, and hepatic or hematologic disorders. In chronic renal failure, itch tends to become more pronounced when hemodialysis is done later than when is done early. Itch may also accompany various obstructive biliary diseases (primary biliary sclerosis, cirrhosis, etc.) in which bile ducts are blocked. If pruritus is less than 1 year, radiological and laboratory tests are necessary to rule out malignant disease. This is especially important for older patients who are susceptible to cancer. In patients with Hodgkin’s disease, a type of malignant hematological tumor, itch may appear months earlier than other systemic symptoms. Itch may also occur in association with intestinal parasites, hyper- or hypothyroidism, diabetes, and acquired immunodeficiency. In addition, medication used in patients with systemic diseases may cause allergic reactions and itch with skin rashes. A detailed history of current and past medication history is essential to detect the possible factors of pruritus.

## 3. Pathophysiology of Itch in Elderly

Several mechanisms were proposed as the pathophysiology of itch in the elderly: dysfunction of cutaneous barrier, cutaneous immunologic reactions, and central and peripheral neuropathy [15].

In addition, multiple cutaneous, systemic, and psychogenic conditions are related to chronic itch in the elderly [15,16].

### 3.1. Dysfunction of Cutaneous Barrier

Dry aging skin, xerosis, is considered to be common cause of itch in elderly patients, with a prevalence ranging from 38 to 85% [17,18,19]. Multiple skin changes the alterations in the barrier function of the stratum corneum (SC), and proteases, pH variations, and decreased activity of sebaceous and sweat glands in the elderly are related to xerosis and chronic pruritus [10,20]. The SC is a barrier to prevent transepidermal water loss and provides protection from external factors. The SC constantly undergoes cellular turnover, and as it ages, the normal process of desquamation can be altered, leading to the appearance of dry skin [21].

The SC is composed of an intercellular lipid matrix (ILM) which maintains the normal barrier function. The ILM is composed of ceramides, cholesterol, and free fatty acids originate from lamellar bodies in the stratum granulosum [22]. Geriatric patients were found to have decreased levels of ILM within the SC. Additionally, the pH of elderly skin becomes more alkaline with age [23]. pH changes may affect enzymatic activity within the SC [24]. As altered enzymatic activity, skin may become dry because of decreased production of natural moisturizing factor, reduced activity of ceramide-forming enzymes [25], and decreased lamellar body secretion [26]. Furthermore, alkaline pH increases the activity of serine proteases in the skin, leading to activation of protease-activated receptor 2 (PAR2) receptors, which induce itch [26,27]. Therefore, changes in pH may induce or exacerbate chronic itch in the elderly population. Other factors that may lead to dry skin include decreased activity of sebaceous and sweat glands [28,29] and decreased levels of hormones, particularly estrogens with alterations in the composition of lipids in women [30]. Thus, skin barrier impairment may cause environment which is vulnerable to exposure of external allergens and irritants. This can lead to allergic contact dermatitis or irritant contact dermatitis in elderly patients with xerosis [31]. In addition, with decreased barrier function, topical medications may cause contact dermatitis and should be prescribed with caution in elderly patients [32].

### 3.2. Cutaneous Immunologic Reactions

The transformation of the immune system result from the process of aging, known as immunosenescence, is related to chronic pruritus. Immunosenescence affects both innate and adaptive immunity, and induces increased levels of autoreactivity [33,34]. Studies reported that bullous pemphigoid (BP), which is more common in the elderly, may manifest with pruritus and a nonspecific urticarial rash accompanied by circulating autoantibodies [35]. Elderly patients suffering from chronic idiopathic pruritus produced evidence of immune dysregulation, such as lymphopenia, eosinophilia, and hypo-gammaglobulinemia. Previous reports suggest that with the progression of immunosenescence, the protective effects of T helper 1 cells are diminishing, which causes higher influence of T helper 2 cell–driven allergic reactions [36]. This immunological imbalance increases the susceptibility of older people to chronic pruritus. In addition, Langerhans cells found in the skin of elderly tend to be decreased dendrites as well as decreased numbers [36].

### 3.3. Central and Peripheral Neuropathy

A previous study suggested that the density of epidermal nerve fibers decreases with age [37]. This phenomenon is also seen in small fiber polyneuropathy. Feng et al. identified an unusual link between age-related loss of Merkel cells, cutaneous touch receptors in skin and alloknesis in elderly skin [38]. They showed targeted genetic deletion of Merkel cells and associated mechanosensitive Piezo2 channels in the skin were sufficient to produce alloknesis [38].

Chronic pruritus in the elderly also can be caused by neuropathic origin. Neuropathic pruritus (NP) can result from central or peripheral nerve damage acquired during the aging process [33,34,39]. The most common sensory ganglionitis known to cause NP is herpes zoster [40]. Herpes zoster is the most common cutaneous viral infection in the elderly [41]. Persistent pruritus following resolution of the primary rash is reported to over 30% [42]. Activation of pruritus-inducing neurons in the affected dermatome is a possible explanation for this form of itch [43]. Diabetes mellitus is the most common cause of small-fiber polyneuropathy in elderly patients and can develop NP [44]. Furthermore, a study found that the presence of diabetes correlates with scalp itch in geriatric patients and scalp itch may be of neuropathic origin [45]. Nerve compression is another cause of chronic pruritus in the elderly [46]. Two forms of radiculopathy related with pruritus in the elderly are brachioradial pruritus (BRP) and notalgia paraesthetica (NP). BRP clinically manifests as pruritus localized in upper extremity, shoulders, neck, back and chest [47]. This form of pruritus usually presents bilaterally and limited to the upper body; however, in rare cases, it may be generalized or unilateral, or it may affect the lower extremities [48]. In addition, in rare cases, central nervous system neurodegenerative disease may produce itching [49].

## 4. Treatment of Itch in Elderly

**Figure 1:**
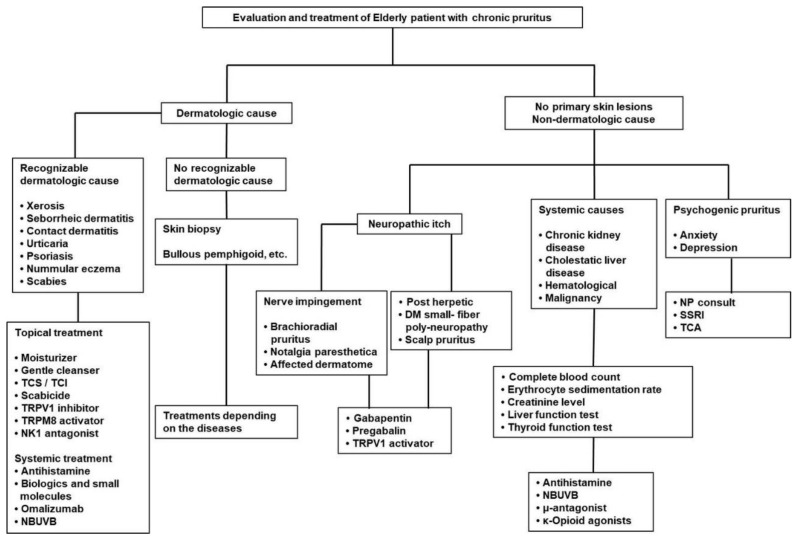
Suggested diagnostic work-up and the treatment for the chronic pruritus in elderly. TCS (topical corticosteroids), TCI (topical calcineurin inhibitor), TRPV (transient receptor potential vanilloid), TRPM (transient receptor potential melastatin), NK (neurokinin), NBUVB (narrowband ultraviolet B), DM (diabetes mellitus), NP (neuropsychology), SSRI (selective serotonin reuptake inhibitor), TCA (tricyclic antidepressant).

If it is suspected that itch is caused by systemic diseases, patients should be asked to provide their detailed medical history and medications. Laboratory examinations such as kidney and liver function tests, should be performed and patients checked for the presence of causative disease, and if present, the causative disease should be treated properly (Figure 1).

### 4.1. Treatment for Dermatologic Causes

#### 4.1.1. Emollient and Gentle Cleanser

Emollients should be suggested as first-line therapy for localized pruritus, patients with chronic kidney disease, and xerosis. Dry skin is caused by changes in the composition of epidermal lipids and increased transepidermal water loss. As the cutaneous barrier function is impaired, hyperkeratosis, erythema, and itching episodes occur [50]. Moisturizers of mixed physiological skin lipids similar to physiological skin lipids (ceramides, cholesterol, fatty acids, etc.) are used to hydrate the stratum corneum, restore the barrier function, and relieve itching [51]. Emollients can contain supplementary ingredients such as urea, polidocanol, menthol, or palmitoylethanolamide with anti-itch properties to target multiple components of the itch pathway [52]. Yet, the fragrances and preservatives in moisturizers can cause the allergic contact dermatitis in some patients who have already itchy dermatitis; hence, the repeat open application test (ROAT) is useful before use. Moreover, since the recovery of the skin barrier function against irritation such as surfactant or alkaline soap is slow for the elderly, a light shower that does not cause severe irritation with a cleanser with mild surfactant is suitable. Xerotic eczema might be worse with frequent visits and stay longin hot places, like saunas.

#### 4.1.2. Topical Corticosteroids and Topical Calcineurin Inhibitors

Topical corticosteroids are effective in treating various cutaneous inflammatory diseases, and reducing inflammation improves the associated pruritus. However, it does not directly control pruritus; hence, its effectiveness may be limited to pruritus without inflammatory skin disease. In addition, the skin barrier function may deteriorate and telangiectasia, senile purpura can occur mainly after long-term use of high potent topical corticosteroid [53].

Topical calcineurin inhibitors are primarily used for inflammatory skin disorders such as atopic dermatitis and seborrheic dermatitis. In addition to their anti-inflammatory effects, they are thought to be effective in reducing pruritus by activating TRPV (transient receptor potential channels) 1 in peripheral C nerve fibers, with subsequent desensitization [54]. Itching will improve within 48 h of the first application, and continued application will continue to reduce itching. Initial stinging due to activation of TRPV1 is a common side effect, but the symptoms of stinging usually improve with repeated application for several days. It is preferable to steroids for long-term use because it has no side effects of skin atrophy even after long-term use [54].

#### 4.1.3. H1-Antihistamines

Oral H1 antihistamines block the H1 receptor on afferent C nerve fibers. They can also inhibit the release of the mediators from mast cells when given in high doses [7]. H1 antihistamines are systemic drugs that are used as the primary treatment in patients with pruritus because of the relative safety, wide availability, and economy of these drugs [54]. However, the data on the effectiveness of systemic antihistamines against itching are limited [55]. So far, data from randomized clinical studies did not prove the effectiveness of antihistamines in diseases other than urticaria [56].

Antihistamines include classic first-generation antihistamines and new second-generation antihistamines. First-generation antihistamines, which include diphenhydramine, chlorpheniramine, and hydroxyzine, easily cross the blood-brain barrier, which leads to sedation and anticholinergic side effects which can cause severe discomfort in the elderly [57]. Anticholinergic side effects include dry mouth, diplopia, visual field disorders, and urinary discomfort. In addition, hydroxyzine is particularly lipophilic and might have a prolonged half-life in elderly patients. The American Geriatrics Society (AGS) Beers Criteria strongly recommend its use in the elderly with caution due to its high anticholinergic activity and risk of delirium and Alzheimer’s disease [58,59]. The newer second-generation antihistamines (e.g., fexofenadine, cetirizine, levocetirizine, loratadine, rupatadine, and ebastine) are recommended as first-line therapy in most dermatologic diseases [60]. These drugs produce less sedation, little anticholinergic activity, fewer drug interactions, and require lower doses compared to first-generation choices.

#### 4.1.4. Immunomodulators

Cyclosporine and azathioprine are effective drugs for inflammatory skin diseases such as neurodermatitis, chronic urticaria, and autoimmune diseases which are hardly affected by antihistamines [61]. The side effects of cyclosporine are high blood pressure, infections, and increased BUN/creatinine, or nephrotoxicity. Nephrotoxicity is often asymptomatic and requires careful monitoring. Azathioprine may cause nausea, vomiting, anemia, hypersensitivity reactions include dizziness, diarrhea, fatigue, and skin rashes. Mycophenolate mofetil has an immunosuppressive effect by specifically blocking lymphocyte proliferation and antibody production. It was reported in severe atopic dermatitis, chronic idiopathic urticaria, and adult autoimmune disease. From a safety point of view, the incidence of toxicity is known to be lower than that of cyclosporine. Methotrexate has an anti-inflammatory effect on lymphocytes and neutrophils and is thought to be effective in treating itching. It was shown to be effective in treating eczema and chronic urticaria. Dapsone was reported to be effective in several types of chronic urticaria and angioedema, but there are side effects such as dose-related anemia, rash, peripheral neuropathy, gastrointestinal side effects, hepatotoxicity, and methemoglobinemia. Due to rare but serious side effects, careful monitoring is necessary.

#### 4.1.5. TRPV1 Inhibitor and TRPM8 Activator

Local neuropathic itching like notalgia paresthetica, brachioradial pruritus, and post-herpes pruritus can be relieved by capsaicin cream, which activates TRPV1 and desensitizes the skin. However, many patients experience a burning sensation after use. Recently, a topical drug that antagonizes and reduces itching rather than activating TRPV1 is currently in clinical trials in patients with atopic dermatitis (PAC-14028, AMOREPACIFIC) [62].

Transient receptor potential M (melastatin) member 8 (TRPM8) is a main channel for temperature-sensitive nerve fibers that are involved in the detection of cooling of body surfaces such as the skin [63]. Activation of TRPM8 relieves itching in various ways [64]. First, it activates the kappa opioid antipruritic receptor [65]. The influx of TRPM8-related calcium into neurons increases the threshold for itching [66]. Calamine or menthol-containing moisturizer relieves the symptoms of itching and makes the skin feel like it is working by activating the channels of the cold receptor (TRPM8 or TRPA1). Cryosim-1, a synthetic substance, acts as a TRPM8 selector, cools the skin without changing the temperature of the tissue, and suppresses symptoms such as itching. It cools in less than a minute and, unlike natural substances like menthol, lasts for 2–4 h. Cryosim-1 showed an immediate improvement of itch symptoms of urticaria when topically applied in a randomized, double-blinded, vehicle-controlled study [67].

#### 4.1.6. Biologics and Small Molecules

The JAK (Janus kinase) inhibitors were developed not only in oral formulations but also in topical formulations. Novel topical therapies such as phosphodiesterase-4 (PDE-4) inhibitors (apremilast, crisaborole) and JAK inhibitors (delgocitinib, tofacitinib, ruxolitinib) are in clinical trials evaluating their efficacy and safety for treatment of skin diseases with pruritus [68,69,70].

Dupilumab is a fully human monoclonal antibody that blocks Interleukin-4 and Interleukin-13 in patients with atopic dermatitis. It was shown to be effective in patients with severe atopic dermatitis and pruritus [71]. In addition to the treatment for atopic dermatitis, the efficacy of dupilumab is confirmed for other diseases with pruritus such as nummular eczema, contact dermatitis, and prurigo nodularis [72,73,74]. Further studies on schedule and dose control for other diseases are needed.

In addition, atopic dermatitis is characterized by a TH2-mediated immune response. Activated TH2 cells in patients have higher IL-31 levels and higher levels of IL-31 in skin. Nemolizumab, which blocks IL-31, markedly diminished pruritus within the first two weeks in patients with atopic dermatitis [72].

Janus kinase (JAK) signaling is involved in signaling of atopic dermatitis related cytokines, such as IL-4, IL-13, IL-31, and IL-17. The JAK inhibitors, targeting different kinases, have distinct mechanisms of action. Neuronal JAK1 activation plays an important role given recently published preclinical and clinical studies [75]. Several Janus kinase inhibitors (baricitinib, upadacitinib, abrocitinib, tofacitnib) are currently undergoing evaluation for efficacy and safety in the treatment of atopic dermatitis [73,74,75,76].

The Neurokinin-1 receptor antagonists (Aprepitant, Tradipitant) are important because they are not only limited to specific diseases but also affect nonspecific pruritus by reducing general itching pathways. They are used for diverse diseases, such as atopic dermatitis, chronic pruritus, and prurigo [77].

The recombinant human monoclonal IgG antibody Omalizumab binds to free IgE and reduces the function of mast cells. Recognizing the clinical efficacy and stability of chronic urticaria, European urticaria treatment guidelines recommend that if urticaria does not respond to antihistamines, cyclosporine should be given priority. As with other treatments, in chronic urticaria, symptoms may slowly recur 4–10 weeks after omalizumab is stopped [61]. Apremilast (PDE4 inhibitor) regulates psoriatic pruritus by directing the production of inflammatory/non-inflammatory cytokines [61].

#### 4.1.7. Ultraviolet Phototherapy

Treatment with ultraviolet (UV)-B, alone or in combination with UV-A, reduces itching caused by chronic kidney disease, and itching in skin conditions such as psoriasis, atopic dermatitis, and other types of eczema are improved. In addition, it can be safely used by patients with underlying illnesses and avoids drug interaction or compliance problems. In these cases, systemic drugs are difficult to use, whereas UV has few side effects other than a temporary sunburn-like reaction [63]. Because the elderly often take medicines for other systemic diseases, UV treatment is often the treatment of choice for pruritus if it is an indication.

### 4.2. Treatment for Neuropathic Itch

#### 4.2.1. TRPV1 Activator

Capsaicin is a substance extracted from pepper that activates TRPV1. Activation of TRPV1 stimulates peripheral nerves associated with itching or pain, releases certain neuroprotein peptides, including substance P, and ultimately causes deficiency. It also induces a continuous desensitization of neurons to various stimuli, which leads to a blockade of the transmission path of pruritus [78]. Capsaicin alleviates the local neuropathic itching such as notalgia paresthetica, brachioradial pruritus, and post-herpes zoster neuropathy. The initial burning sensation, which can last for several days, is a major side effect [79]. Applicating local anesthesia first and then capsaicin for two weeks at the start of treatment can reduce the burning sensation. A recently developed 8% capsaicin patch only requires a single application [80].

#### 4.2.2. Neurological Drugs

The structural analogues of the neurotransmitter γ-aminobutyric acid (i.e., gabapentin and pregabalin) are effective against various types of itching [56]. The mechanism of action is unclear, but these appear to be effective in reducing sensitization of the central nervous system and thus, are effective in chronic pruritus. In a controlled study in patients with pruritus due to chronic kidney disease, low doses of gabapentin (100–300 mg three times a week) were much more effective than placebo in reducing pruritus [81]. Although there are no controlled studies, one case was reported of the use of these drugs to reduce neuropathic pruritus such as brachioradial pruritus [68]. Gabapentin is also effective for pruritus of chronic kidney disease [82]. Side effects include drowsiness, weight gain, ataxia, leg swelling, blurred vision, and constipation. In addition, pregabalin may induce withdrawal symptoms such as diaphoresis, tachycardia, hypertension, tremors, diarrhea, agitation, paranoia, auditory hallucinations, and suicidal attempt if stopped abruptly. Therefore, cessation of medication should be tapered [56].

### 4.3. Treatment for Itch Due to Systemic/Psychogenic Causes

The most important thing in pruritus caused by systemic disease is the management of the underlying disease above all else. For example, in iron deficiency anemia, iron medications are prescribed, and in hypothyroidism, thyroid hormones are supplied. In chronic kidney disease-associated pruritus, appropriate treatment such as hemodialysis is provided, and in cholestatic pruritus, biliary tract disease is first treated, while itching can be controlled with cholestyramine. Moisturizers and non-sedative antihistamines can be prescribed for all pruritus.

#### 4.3.1. Opiate Agonists and Antagonists

Opiate agonists and antagonists are effective against cholestasis, chronic urticaria, atopic dermatitis, and chronic kidney disease. Randomized controlled trials showed the antipruritic effect of mu-opioid antagonists (naltrexone, nalmefene, naloxone) in patients with chronic urticaria, neurodermatitis, eczema, and cholestasis [56]. They have side effects such as nausea, loss of appetite, stomach cramps, and diarrhea. In addition, a randomized, placebo-controlled clinical trial showed that the kappa-opioid agonist nalfurafine hydrochloride significantly reduces itching in patients with chronic kidney disease [51]. Kappa opioid analogs and mu opioid antagonists (butorphanol) were reported to reduce itching associated with non-Hodgkin’s lymphoma, cholestasis, and other refractory pruritus [83].

#### 4.3.2. Antidepressants

Selective serotonin reuptake inhibitors (paroxetine, sertraline, fluvoxamine, and fluoxetine) were reported to reduce various types of general pruritus in addition to psychogenic itching [84]. Side effects include anticholinergic effects and sexual dysfunction. Tricyclic antidepressants such as amitriptyline and doxepin are sometimes used to treat chronic pruritus, but there are no randomized studies [50]. Elderly patients are particularly susceptible to the anticholinergic side effects of this agent, such as urinary retention, constipation, dizziness, dry mouth, cardiac conduction abnormalities, and blurred vision.

## 5. Conclusions

Pruritus in the elderly papulation is a common symptom that occurs not only in skin diseases but also under a variety of other circumstances, such as secondary, systemic, or psychotic diseases. Elderly patients with itching suffer from extreme distress and poor quality of life without proper treatment. The factor stimulating the itch and the extent of the symptoms affect the treatment. Various treatments are used to relieve itching, but data are too limited to directly compare many studies on the effectiveness of these treatments. Many novel drugs have been developed for itching and may be useful if used appropriately according to the specific condition of an individual.

## Figures and Tables

**Table 1:** Causes and characteristics of pruritus in various diseases in elderly [7].

Classifications	Diagnosis	Clinical Manifestation of Pruritus
Cutaneous diseases	Dry skin (xerosis)	With flare-ups at dry climate
	Irritant and allergic contact dermatitis	Mainly limited to the skin lesion
	Seborrheic dermatitis	Mainly limited to the skin lesion
	Atopic dermatitis	Scratching exacerbates pruritus Accompanied by allokinesis, stinging, burning sensation
	Psoriasis	usually limited to the skin lesions
	Urticaria	Mechanically induced by such as tight clothing Accompanied by wheal, and flare
Unknown	Senile Pruritus	without a primary rash the absence of xerosis or other recognizable causes
Systemic diseases	Chronic kidney disease	2–3 months after dialysis Accompanied by xerosis or prurigo Generalized or localized
	Hepatobiliary diseases	Can be mechanically induced Not diminished by scratching Usually generalized pruritus
	Thyroid disorders	Hyperthyroidism/hypothyroidism Associated with urticaria
	Polycythemia vera	After contact with water Accompanied by stinging sensation and prurigo Generalized pruritus
	Iron deficiency anemia	Generalized pruritus Skin lesion or irritation provokes scratching
	Hodgkin’s lymphoma	Premonitory onset Area of affected lymph nodes such as mediastinal sites
Drug-induced pruritus	Drug-induced pruritus Drug eruption	With or without skin rash Can occur after several months (lichenoid type)
Neurological disorders	Postherpetic neuralgia	With painful qualities such as burning, stinging
	Brachioradial pruritus	Triggered by UV light Brachioradialis muscle (C6 dermatome) Unilateral or bilateral
	Notalgia paraesthetica	Hyperpigmented lesions Between the scapula or on the back
Psychiatric disorders	Somatoform disorders, dissociative disorders, schizophrenia	Sometimes severe lesions with scratching With painful qualities such as burning, stinging From the head to the trunk or whole body
	Hallucinations, delusional parasitosis	Symptoms of a bug crawling Particles are collected as evidence
	Adjustment disorder	Accompanied with depression or other psychosomatic symptoms

## Abbreviations

TSLP	Thymic stromal lymphopoietin
IL	Interleukin
PAF	Platelet activator factor
ILC	Innate lymphoid cell
GABA	γ-aminobutyric acid
TRPV	Transient receptor potential vanilloid
TRPA	Transient receptor potential ankyrin
TRPM	Transient receptor potential melastatin
GPCR	G-protein-coupled receptor
JAK	Janus kinase
ROAT	Repeat open application test
GRADE	Grading of Recommendations, Assessment, Development and Evaluation
EMLA	Eutectic mixture of local anesthetics
TCA	Tricyclic antidepressant
5-HT	5-hydroxytryptamine
SSRI	Selective serotonin reuptake inhibitor
SNRI	Selective serotonin and norepinephrine reuptake inhibitor
NK	Neurokinin
DM	Diabetes mellitus
NP	Neuropsychology
